# Influence of Aortic Arch Morphology on Renal Perfusion in Patients with Coarctation of the Aorta: An Exploratory Study

**DOI:** 10.3390/medicina60060886

**Published:** 2024-05-28

**Authors:** Sigitas Cesna, Augustinas Bielinis, Tadas Zvirblis, Marius Miglinas, Virgilijus Tarutis

**Affiliations:** 1 Clinic of Cardiac and Vascular Diseases, Institute of Clinical Medicine, Faculty of Medicine, Vilnius University, M.K. Čiurlionis str. 21, LT-03101 Vilnius, Lithuaniavirgilijus.tarutis@santa.lt (V.T.); 2 Vilnius University Hospital Santaros Klinikos, Santariskiu str. 2, LT-08406 Vilnius, Lithuania; 3 Department of Human and Medical Genetics, Institute of Biomedical Sciences, Faculty of Medicine, Vilnius University, LT-03101 Vilnius, Lithuania; 4 Institute of Data Science and Digital Technologies, Faculty of Mathematics and Informatics, Vilnius University, LT-03225 Vilnius, Lithuania

**Keywords:** coarctation of the aorta, arterial hypertension, blood pressure, renal perfusion scan, renal scintigraphy

## Abstract

*Objectives*: The configuration of the aortic arch, particularly a Gothic arch shape, in individuals with corrected coarctation of the aorta (CoA) has been associated with a decreased systolic wave amplitude across the arch, which could potentially impair renal perfusion and elevate the risk of arterial hypertension. This study aims to explore the relationship between the morphological characteristics of the aortic arch and their impact on renal perfusion in patients with CoA. *Methods*: Seventy-one subjects with corrected CoA underwent continuous 24 h ambulatory blood pressure monitoring, computed tomography to assess the aortic arch, and renal perfusion scanning. Subjects were stratified into three groups based on the height-to-width (H/W) ratio of their aortic arch: Group 1 with a H/W ratio of <0.65, Group 2 with a H/W ratio between 0.65 and 0.85, and Group 3 with a H/W ratio of >0.85. *Results*: Groups 1 and 2 (53,78% and 62.63%) presented with a higher hypertension prevalence of elevated blood pressure than Group 3 (38.89%). Notable variations were observed among the subjects in the time to peak perfusion (Tmax) in the left kidney across the groups. Group 1 showed a median T_max_ at 0.27, Group 2 at 0.13, and Group 3 at −0.38 (*p*-value = 0.079). The differences in T_max_ for the right kidney followed a similar trend but were not statistically significant (Group 1 at 0.61, Group 2 at 0.22, and Group 3 at 0.11; *p*-value = 0.229). *Conclusions*: This study suggests that variations in the aortic arch morphology might not significantly influence renal perfusion in CoA patients. This indicates the potential adaptability of the renal blood flow, which appears to compensate for reduced perfusion, thus minimizing adverse effects on the kidney function. This adaptability suggests an inherent physiological resilience, emphasizing the need for further targeted research to understand the specific interactions and impacts on treatment strategies for CoA.

## 1. Introduction

Coarctation of the aorta (CoA) represents a prevalent congenital cardiac anomaly, ranking fifth most common [1,2,3,4,5]. This condition is characterized by a constriction in the aorta, leading to increased blood pressure in the upper extremities and regions proximal to the constriction while simultaneously reducing blood flow distal to the narrowing. Historical data indicate a grim prognosis for untreated CoA, with a mortality rate of 25% before the age of 20 during the period from the 1930s to the 1960s. Moreover, the mortality rate escalated from 1.6% in the initial two decades to 6.7% annually in the sixth decade and beyond [6].

Consequently, early detection and prompt surgical or interventional correction are imperative to prevent the onset of persistent or residual arterial hypertension (AH) [7]. Notwithstanding successful early interventions, there remains a substantial risk for the development of cardiovascular pathologies, with systemic AH persisting in approximately 60% of individuals post surgery or percutaneous treatment [6,8]. The predominant phenotype observed in CoA patients is isolated systolic hypertension [9]. Notably, some individuals may be classified as hypertensive, even in the presence of normotensive blood pressure readings, if they are undergoing antihypertensive therapy [10].

The persistence of hypertension in patients with post-repair CoA is multifactorial, involving residual narrowing; aberrant arch geometry; endothelial dysfunction; altered arterial smooth muscle reactivity; modifications in the aortic wall, contributing to increased arterial stiffness; and diminished baroreceptor sensitivity, and culminates in elevated sympathetic nervous system activity [11,12,13,14,15]. A post-surgical Gothic arch configuration is linked to reduced distensibility of the ascending aorta and a notable reduction in the systolic wave amplitude across the arch compared to individuals possessing a standard arch configuration [14]. A Gothic arch is characteristically taller and narrower than the wide and shallow form of crenel-type aortic arches, where the height-to-width ratio is lower.

This study represents a novel exploration of the correlation between the morphological attributes of the aortic arch and their repercussions on renal perfusion. The underlying hypothesis posits that distinct anatomical configurations of the aorta might impact renal functionality through the sustained activation of the renin–angiotensin–aldosterone system. This physiological mechanism parallels the pathophysiological processes observed in renovascular disease, wherein captopril renal scintigraphy is employed as a diagnostic tool to detect such conditions [16,17]. Renal scintigraphy has a high diagnostic accuracy for renal artery stenosis and renovascular hypertension, as evidenced by sensitivity rates ranging from 87% to 96% and specificity rates from 85% to 95% [16,18].

## 2. Materials and Methods

The current study received approval from the Vilnius Regional Biomedical Research Ethics Committee (2019/5-1113-619). All the participants provided informed consent, as evidenced by their signatures on the patient consent forms. The authors are fully accountable for the conceptualization, execution, and analytical methodologies of the study, as well as for the composition, revision, and finalization of the manuscript’s content.

Study population

**Figure 1:**
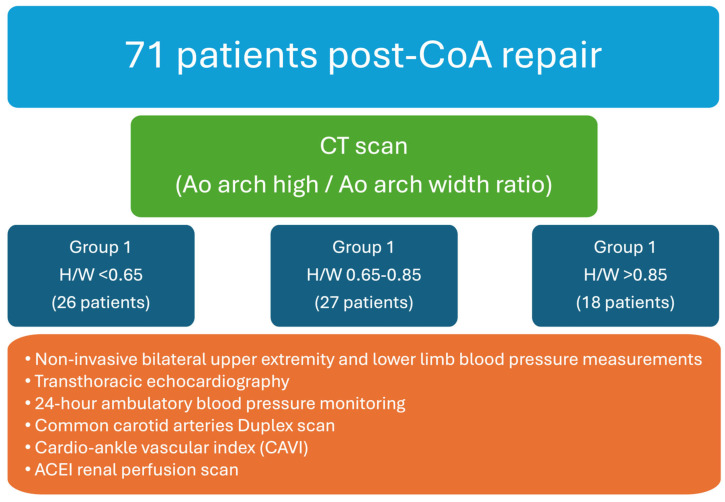
Research design.

In this prospective cohort study, adult individuals with post-repair (surgical or percutaneous) coarctation of the aorta were enrolled. The exclusion criteria encompassed individuals with complex congenital heart diseases; hemodynamically significant aortic valve stenosis; chronic renal insufficiency, characterized by an estimated glomerular filtration rate (eGFR) of <45 mL/min; patients diagnosed with stage III-IV malignancies or psychiatric disorders; and those who were pregnant or intending to conceive during the study period. Participants enrolled in the study underwent an initial clinical evaluation followed by an extensive assessment regimen. This regimen included continuous 24 h ambulatory blood pressure monitoring, common carotid artery duplex scans, calculation of the cardio–ankle vascular index (CAVI), delineation of the aortic anatomy through computed tomography (CT) imaging, and evaluation of the renal function via renal perfusion scanning (Figure 1).

Diagnostic workup.

During the initial consultation, a comprehensive clinical history was compiled for each patient, encompassing prior surgical procedures, interventions, comorbidities, and medication regimens. This preliminary evaluation was succeeded by bilateral upper extremity and lower limb blood pressure (BP) measurements. Transthoracic echocardiography was conducted for all the participants to assess the left ventricular mass and function and the pressure gradient across the aortic arch. Furthermore, ambulatory 24 h blood pressure monitoring was implemented, with the cuff strategically positioned on the right arm, adhering to established guidelines for individuals diagnosed with CoA [19]. Arterial hypertension was defined as a mean daytime systolic blood pressure (SBP) of ≥140 mmHg or a diastolic blood pressure (DBP) of ≥90 mmHg, as measured using ambulatory blood pressure monitoring (ABPM), or the patient was on antihypertensive medication.

**Figure 2:**
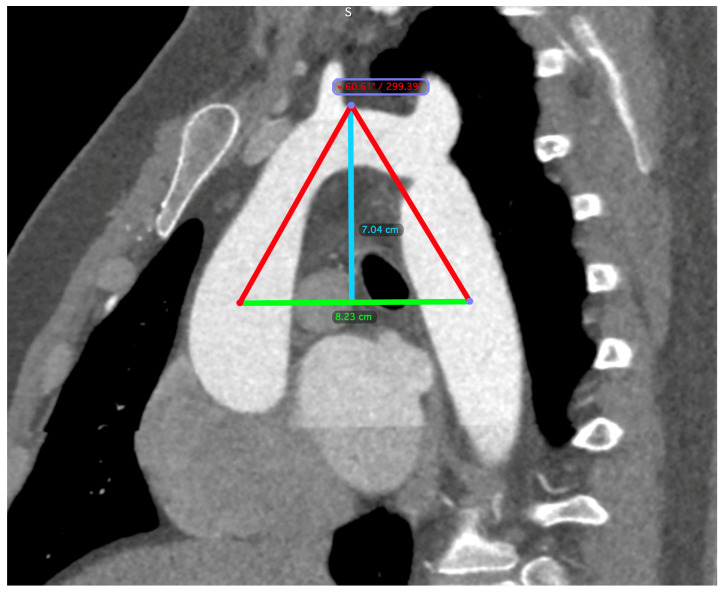
CT scan measurements. A triangle is formed between the apex of the arch (indicated by the blue line) and the midpoints of the ascending and descending aorta (indicated by the green line), which are aligned with the right pulmonary artery. The arch angle was defined as the vertex angle of the resultant triangle, and the arch width was the basal length of this triangle. The perpendicular distance from the basal length to the arch’s apex determined the arch height.

The diagnosis of CoA was established via computed tomography (CT) imaging. Aortic dimensions were meticulously determined through three-dimensional reconstructions, employing oblique angles orthogonal to the vessel’s longitudinal axis. The aortic perimeter was calculated using the formula: perimeter divided by π (where π is approximately 3.14). Measurements were taken at critical anatomical points, including the midpoints of the ascending aorta and descending aorta, both aligned with the right pulmonary artery, the midpoint of the aortic arch (either situated between the common carotid artery and the left subclavian artery or located at the apex of the aortic arch), the isthmus, and the aorta at the diaphragmatic level. A triangle was constructed, linking the arch apex with the midpoints of the ascending and descending aorta, from which the arch angle (vertex angle of the triangle) and arch height and width (base of the triangle) were derived. The arch height was ascertained by the perpendicular distance from the base to the highest midpoint of the arch (Figure 2).

**Figure 3:**
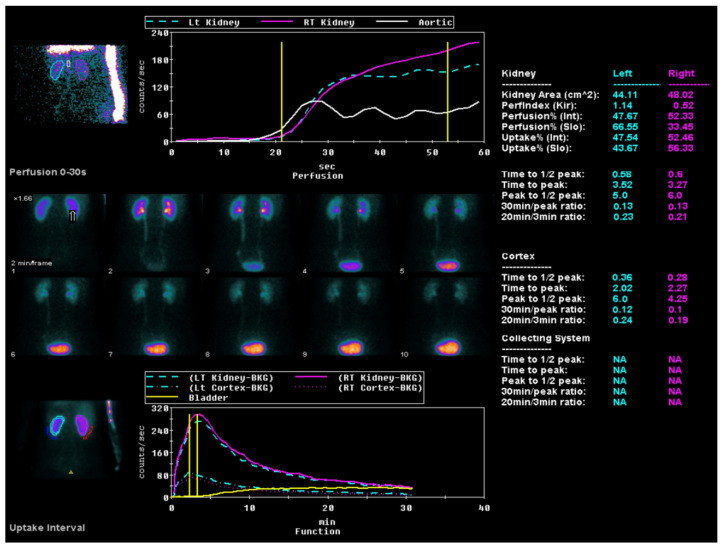
ACE inhibitor renography using the radiopharmaceutical 99 mTc-MAG3.

All the study participants underwent ACE inhibitor renography using the radiopharmaceutical 99 mTc-MAG3 (Figure 3) by the consensus report on ACE inhibitor renography guidelines to identify renovascular hypertension [20]. All the patients were instructed to discontinue their ACE inhibitor medication three days before the scan to prevent potential interference with the results. Renal scintigraphy analysis involved the generation of time–activity curves by designating a region of interest (ROI) over each kidney, including the background area, and delineating the aorta. Renal perfusion assessment entailed visual and quantitative analyses of the initial radiopharmaceutical bolus as it passed through the abdominal aorta and into the renal arteries. The relative renal function was evaluated based on the time–activity curve data, focusing on the relative radiopharmaceutical uptake. The T_max_, or time to the peak, was calculated from the radiopharmaceutical injection moment to the peak of the renogram curve. The 30 min/peak ratio, indicative of the renal transit time and parenchymal function, was determined by comparing kidney counts at 30 min post injection to peak counts obtained during the scintigraphy. The T½, or time to the half-peak, was measured from the peak counts to when the renogram curve reduced to half its maximum. Furthermore, the ratio of the whole kidney ROI counts at 20 min to those at 3 min (20 min/3 min ratio) was computed, serving as an essential metric for concurrently evaluating renal clearance and excretion [21]. All the diagnostic evaluations were completed within two months, ensuring a minimum interval of two weeks between each patient’s CT and renal perfusion scans.

The perfusion graph shows the tracer flow through the left and right kidneys and the aorta over time, charted against count rates. A sequential series highlights the tracer distribution in the kidneys during the first 30 s post injection. The uptake graph illustrates longer-term tracer uptake and excretion, focusing on kidney and bladder activity. Quantitative data provide detailed metrics, such as the kidney area, perfusion indices (T_max_, T½, 30 min/peak ratio, and 20 min/3 min ratio) uptake percentages, and peak times, for both kidneys.

**Statistical analysis.**

Descriptive statistical analysis, such as mean (and standard deviation (SD)) or, otherwise, median with (Q1 and Q3), were used for continuous variables. Frequency distributions between groups were compared using Fisher’s exact test based on the expected frequencies. The normality of the continuous variables was assessed by the Shapiro–Wilk test. Most variables were distributed non-normally, so we used the Kruskal–Wallis test to evaluate the medians’ differences between independent groups. We considered the differences as being statistically significant when the *p*-value was < 0.05.

## 3. Results

This comprehensive study, conducted from 2019 to 2022, encompassed a cohort of 71 subjects with residual coarctation of the aorta. All the participants were rigorously selected based on predetermined inclusion and exclusion criteria. Table 1 offers an exhaustive delineation of this patient population’s demographic and clinical characteristics. The subjects were methodically categorized into three distinct echelons predicated on the aortic arch’s height-to-width (H/W) ratio: Group 1 included individuals with an H/W ratio of less than 0.65, Group 2 constituted those with a ratio between 0.65 and 0.85, and Group 3 was reserved for those exceeding an H/W ratio of 0.85.

Within the research cohort, the distribution of participants was as follows (Table 1): Group 1 comprised 36.62% of the cohort, with 26 individuals; Group 2 constituted 38.03%, with 27 individuals; and Group 3 made up 25.35%, with 18 individuals. The analysis of the age disparities at the point of the study enrolment and during the initial CoA repair revealed no statistically significant differences across the groups. Furthermore, the median (Q1–Q3) age of the patients at the time of the study was 27.66 years (23.26–36.87) for Group 1, 28.03 years (23.1–35.29) for Group 2, and 32.33 years (22.16–38.57) for Group 3, with these differences also not reaching statistical significance.

Additionally, Group 1 was characterized by a higher proportion of male participants, accounting for 69.22%, compared to 55.66% in Group 2 and 38.99% in Group 3. Despite this disparity in the gender distribution, the difference did not achieve statistical significance, with a calculated *p*-value of 0.146. Notably, a significant distinction was observed in the body surface area (BSA) across the groups, with Group 1 having a notably larger median BSA (2.01 (1.91–2.23)) relative to Group 2 (1.87 (1.74–2.04)) and Group 3 (1.88 (1.61–1.99)), as evidenced by a *p*-value of 0.002. The median age of the patients at the time of their initial CoA repair was observed to be the highest in Group 2, recorded at 2815 days (with an interquartile range from 623 to 5167), whereas, in Group 1 and Group 3, the median ages were 2222 days (175–3531) and 1723 days (648–3984), respectively (*p* = 0.577). The surgical approach predominantly employed across all the groups was the end-to-end anastomosis, suggesting uniformity in the surgical technique applied among the study participants.

The study further revealed pronounced morphological variations within the aortic arch dimensions, precisely, the height, width, and angle, when patients were stratified by their H/W ratio. These significant morphological distinctions suggest variability in the aortic arch structure associated with differing H/W ratio subgroups. Conversely, no substantial differences were discerned in parameters such as residual stenosis and the growth index across the groups, indicating that these factors are potentially influenced by determinants other than the H/W ratio.

During the 24 h ambulatory blood pressure monitoring, a distinct pattern was observed regarding hypertension prevalence across the stratified groups (Table 2). Notably, patients in Group 1 and Group 2 demonstrated elevated mean systolic blood pressure measurements in the right arm (143.5 mmHg and 139 mmHg, respectively), in contrast to Group 3 (Gothic arch), where the mean systolic blood pressure was recorded at 129.5 mmHg. This trend was also reflected in administering antihypertensive medications: 61.11% of the patients in Group 3 were not on any antihypertensive therapy compared to 46.22% in Group 1 and 37.37% in Group 2. Despite these variations, the statistical analysis of the antihypertensive medication utilization did not yield any statistically significant differences, as evidenced by a *p*-value of 0.868. Additionally, the left ventricular mass index (LVMi) was elevated in Group 1, with an average value of 90.99 g/m^2^. This contrasts with the LVMi values in Group 2 and Group 3, which were 86 g/m^2^ and 81.22 g/m^2^, suggesting a possible augmentation in cardiovascular strain within these groups.

The pulse wave velocity (PWV) exhibited typical values without significant variation across the groups, and no statistically significant differences were found in the cardio–ankle vascular index (CAVI) measurements on either side. Similarly, the carotid artery stiffness remained consistent across all the groups, showing no statistically significant differences. However, higher carotid artery wall strain on both sides was associated with the crenel-type aortic arch (Group 1), although this did not reach statistical significance.

Within the defined patient cohort, a higher frequency of left vertebral artery (LVA) hypoplasia was noted in Group 3 relative to Groups 1 and 2. In particular, LVA hypoplasia was identified in 50% of the patients in Group 3 compared to 15.3% in Group 1 and 37.03% in Group 2. Although there was a noticeable trend in the distribution of this anatomical variation, the differences observed did not reach a level of statistical significance.

Renal perfusion assessments, as detailed in Table 3, conducted across the three groups post-captopril administration, did not reveal any statistically significant disparities. However, both kidneys in Group 1 had the most remarkable median change in renal uptake, although this did not reach statistical significance (*p* = 0.308). Furthermore, the median alteration in the time to the half-peak (T½ change) was higher in Group 1 but remained statistically non-significant across all the groups for both the left and right kidneys.

A significant variation in the time to the peak (T_max_) was observed in the left kidney among the groups. Group 1 exhibited a median T_max_ of 0.27, Group 2 had a median of 0.13, and Group 3 presented a median of −0.38, with a *p*-value of 0.079. Although a similar trend was noted for the right kidney, the difference was not statistically significant: Group 1 had a median T_max_ of 0.61; Group 2 had a median of 0.22; and Group 3 had a median of 0.11, yielding a *p*-value of 0.229.

There were no statistically significant differences in the T_max_/T½ and 30 min/T_max_ ratios for both kidneys across the groups. However, a negative median change was noted in the 20 min/3 min ratio for the right kidney in Group 3, which approached statistical significance (*p*-value = 0.052).

## 4. Discussion

In the present study, we initially hypothesized that variances in the aortic arch morphology might contribute to differential renal hypoperfusion levels, potentially influencing residual arterial hypertension in patients. A prospective analysis investigated this hypothesis, involving individuals presenting with residual aortic coarctation.

The association of a Gothic arch configuration, characterized by a marked increase in arch height relative to its width, with post-CoA repair outcomes has been widely recognized. There is speculation among researchers that a truncated aortic isthmus might lead to a predisposition toward an angulated, or Gothic, arch configuration following CoA repair [22]. Moreover, the role of surgical techniques in potentially influencing the development of a Gothic arch configuration is a subject of ongoing debate [23]. Interestingly, a significant correlation has been observed in various studies where patients displaying a Gothic arch configuration predominantly underwent extended aortic arch anastomosis. This procedure results in an expanded aortic dimension between the left subclavian and carotid arteries, leaving the segment between the brachiocephalic and left carotid arteries unaffected [8]. Contrary to potential expectations, our study found that most participants underwent end-to-end anastomosis, with no notable difference observed across the different groups regarding the surgical technique employed.

Early intervention in CoA repair appears to preserve the elasticity of the conducting arteries, a factor partially elucidating the established correlation between the timing of the repair and patient prognosis. However, concerns persist regarding functional abnormalities at the endothelium level and within smooth muscle cells following early repair, underscoring the necessity for meticulous, prolonged surveillance of patients undergoing CoA repair [24]. Our study contributes to this body of knowledge by revealing that the median age at the time of the CoA repair was below six years across all the groups, supporting the practice of early surgical intervention. Additionally, our analysis indicated a distinct pattern in which patients exhibiting a crenel arch morphology were more likely to present with hypertension and require antihypertensive treatment compared to those with a Gothic arch morphology. This observation was further substantiated by the data, which showed a higher left ventricular mass index and a greater prevalence of antihypertensive medication use among patients with a crenel arch, suggesting a more pronounced cardiovascular burden in this group.

Survivors of CoA repair in young adulthood, exhibiting an angulated Gothic aortic arch upon follow-up, demonstrated early and heightened systolic wave reflection alongside increased aortic stiffness compared to those with a more conventionally smooth and rounded aortic arch configuration [25]. Despite these findings, our study’s outcomes did not align with the expected increase in aortic stiffness among the Gothic arch group. When evaluating parameters such as pulse wave velocity (PWV), arterial compliance, and distensibility, we observed no significant discrepancies among the groups differentiated by their aortic arch morphologies. This absence of significant findings could be attributed to the constrained sample size of our subgroups, which may limit the statistical power necessary to detect subtle differences in aortic stiffness and related vascular parameters.

Contrary to our anticipations, the study’s findings indicated an absence of renal perfusion disorder among patients with either native or repaired CoA, as none of the groups met the predefined criteria for assessing alterations in renal perfusion disruption [21]:

1. a change in the 30 min/peak uptake ratio of 0.15 or greater;
2. an increase in T_max_ of at least 2 min or 40%;
3. a greater-than-10% change in the relative uptake after ACE inhibition.

This revelation challenges the previously assumed correlation among the severity of the aortic coarctation, aortic arch morphology, and renal perfusion deficits, suggesting that the mechanisms underlying residual hypertension post-coarctation treatment might be more intricate and less directly associated with renal perfusion than previously considered.

Interestingly, our study noted a significant occurrence of left vertebral artery hypoplasia in half the patients within the group characterized by a Gothic arch configuration (5050%). Vascular anomalies, such as vertebral artery hypoplasia and an incomplete posterior circle of Willis, have been associated with increased cerebral vascular resistance. The heightened prevalence of these anomalies among patients with aortic coarctation, as compared to the general population, highlights a potential independent risk factor for hypertension. This finding suggests an additional dimension to the cardiovascular challenges faced by individuals post-coarctation repair, potentially linking vascular anomalies to an increased risk of stroke and hypertension within this patient cohort [26]. However, it is essential to note that our study’s results did not definitively confirm the proposed relationship between these cerebral vascular anomalies and the observed clinical outcomes in aortic coarctation patients. This discrepancy underscores the complexity of cardiovascular pathophysiology in the aftermath of coarctation repair.

The LV mass index is significantly elevated in patients with a Gothic arch compared to those with a crenel-type arch, and a positive correlation was noted between the LV mass index and indices of the systolic wave reflection, central aortic stiffness, and systolic BP [25]. Specifically, we observed an elevation in the LV mass index in patients with a crenel arch configuration of the aortic arch compared to those with a Gothic-type arch. This increase in the LV mass index, indicative of cardiac remodeling, was positively correlated with indices of the systolic wave reflection, central aortic stiffness, and systolic BP, suggesting that patients with a crenel arch configuration might be at a higher risk for developing cardiovascular complications.

Additionally, our analysis showed that patients with a crenel-type aortic arch were predominantly male, had a larger body surface area, and displayed slightly higher rates of elevated blood pressure than their counterparts. This observation aligns with the existing literature, suggesting a potential link between a larger body surface area or obesity and increased blood pressure levels. The interplay among body size, aortic arch morphology, and cardiovascular health underscores the multifactorial nature of hypertension and cardiac remodeling post-CoA repair [27].

Our study builds upon prior research indicating that patients with a Gothic arch morphology experience reduced ascending aorta distensibility and increased systolic wave amplitude loss across the aortic arch. Such findings suggest a mechanistic explanation for the development of arterial hypertension in these patients, even without residual arch stenosis. These alterations in hemodynamic parameters hint at a potentially intrinsic property of the Gothic arch shape that predisposes hypertension by affecting the aorta’s physiological function [14].

In contrast, our observations revealed that patients with a crenel-type aortic arch presented with higher T_max_ changes after ACE inhibition in renal perfusion, indicative of altered renal hemodynamics. This suggests that wider aortic arches associated with the crenel morphology might impede the renal flow, thereby contributing to the development of arterial hypertension. This differentiation in the impacts of the aortic arch morphology—hemodynamic influences on baroreceptors in Gothic arches versus influences on renal flow in crenel-type arches—proposes an intriguing area for further investigation. The hypothesis that the Gothic arch morphology exerts a more pronounced hemodynamic influence on the cardiovascular system, potentially affecting the baroreceptor sensitivity and function, while crenel-type arches primarily impact the renal flow, underscores the complex interplay among the vascular structure, renal hemodynamics, and hypertension. This complexity necessitates more comprehensive studies to elucidate the specific mechanisms through which different aortic arch morphologies impact blood pressure regulation.

The prolongation of T_max_ and T½ in Group 1 suggests that the crenel-type aortic arch may contribute to slower renal perfusion and excretion. Although statistical significance was not achieved, a comparison of the absolute values between the baseline and post-ACEI T_max_ and T½ indicates a discernible pattern that supports this inference. This trend could imply differential renal function responses between the groups, which might be substantiated in more extensive future studies.

Our study’s findings, which did not identify the anticipated alterations in renal perfusion across different aortic arch morphologies, cast new light on the understanding of cardiovascular–renal interactions. The lack of significant deviations in renal perfusion parameters suggests that the impact of the aortic arch morphology on renal blood flow and, by extension, on systemic blood pressure regulation may not be as profound as previously considered. This observation might be explained by the kidney’s inherent capacity to adapt to variations in hemodynamic pressures, ensuring renal perfusion is maintained, even under potentially compromised arterial flow conditions.

Observing sustained elevated blood pressure and enhanced blood flow to the neck vessels post-aortic coarctation repair hints at the complex multifactorial nature of residual hypertension in this patient population. This phenomenon may not solely be attributable to the anatomical correction of the coarctation but also to functional changes within the cardiovascular system that persist post repair. Factors such as diminished baroreceptor sensitivity, decreased arterial wall elasticity, and increased arterial stiffness could significantly contribute to the persistence of hypertension despite successful CoA repair. These elements underscore the intricate interplay between structural corrections and the functional adaptations of the cardiovascular system.

The parallel drawn with renal artery stenosis, where interventional procedures, like stenting, may not fully address the multifaceted aspects of hypertension, reinforces the notion that a similar complexity exists in the management of hypertension post-CoA repair. This comparison serves as a critical reminder of the limitations of mechanical interventions alone in addressing the functional and adaptive changes that contribute to hypertension.

Consequently, this insight calls for a holistic approach for managing arterial hypertension in CoA patients, emphasizing the anatomical correction and the functional and adaptive aspects of the cardiovascular system. Such an approach would entail a thorough evaluation of patient-specific factors, including the timing of the intervention; the choice between surgical repair or interventional cardiology techniques, like stenting; and the integration of medical therapy to address the multifactorial nature of hypertension in this context.

## 5. Conclusions

Our exploratory study suggests that variations in the aortic arch morphology may not significantly impact renal perfusion in patients with coarctation of the aorta. This observation underscores the potential adaptability of the renal blood flow, which compensates for reduced perfusion, thereby minimizing adverse effects on kidney function. However, these findings are derived from a small pilot study and, thus, need more statistical power to be universally applied to all CoA patients. Consequently, the generalizability of these observations to a broader CoA population remains to be determined.

Given that our results do not support the initial hypothesis, it is premature to advocate for scaling this research to larger-scale studies without additional exploratory work. Further targeted investigations are needed to examine specific factors that could influence outcomes in CoA treatment. Such studies would be crucial in refining our understanding of when and how interventions, like stenting, could be the most effective in managing complex hypertension cases associated with CoA.

As this field of research progresses, it remains essential to meticulously examine the intricate relationships among the aortic arch structure, renal perfusion dynamics, and overall cardiovascular health in CoA patients. By focusing on these detailed aspects, future research can ensure that treatment strategies are not only based on anatomical correction but also precisely tailored to the physiological responses of individual patients, optimizing treatment outcomes.

## 6. Study Limitations

This study’s limitations include a small sample size because of the relatively limited population of patients with aortic coarctation. Although statistical significance was not reached, a discernible pattern supports this inference, which could be validated in more extensive future studies. Furthermore, the diagnostic procedures were inconsistent, as not all the patients underwent identical instrumental or blood tests.

## Figures and Tables

**Table 1:** Patient population’s demographic and clinical characteristics.

	Group 1 (H/W < 0.65) n = 26 Median (Q1–Q3)	Group 2 (H/W 0.65–0.85) n = 27 Median (Q1–Q3)	Group 3 (H/W > 0.85) n = 18 Median (Q1–Q3)	*p*-Value
Patients	26 (36.62)	27 (38.03)	18 (25.35)	
Male, n (%)	18 (69.22)	15 (55.66)	7 (38.99)	0.146
Age, years	27.66 (23.26; 36.87)	28.03 (23.1; 35.29)	32.33 (22.16; 38.57)	0.659
Age at first operation, days	2222 (175; 3531)	2815 (623; 5167)	1723 (648; 3984)	0.577
BSA	2.01 (1.91; 2.23)	1.87 (1.74; 2.04)	1.88 (1.61; 1.99)	0.002
Operation type, n (%)				
End-to-end anastomosis	24 (92.3)	20 (74.1)	17 (94.4)	0.422
Gore-Tex	1 (3.8)	-	-	
Homotransplant	1 (3.8)	1 (3.7)	-	
Pericardial patch	-	1 (3.7)	1 (5.6)	
Subclavian patch	-	1 (3.7)	-	
Stent	-	4 (14.8)	-	
CT scan measurements				
Ao arch height, mm	39 (36.4; 42.9)	46.9 (42.5; 52.4)	53.55 (51.7; 58.1)	<0.001
Ao arch width, mm	68.35 (59.9; 81.5)	62.2 (60.4; 67.9)	56.85 (54; 59.2)	<0.001
Ao arch height/Ao arch width	0.58 (0.51; 0.61)	0.73 (0.69; 0.78)	0.95 (0.91; 0.99)	<0.001
Ao arch angle, degrees	76.42 (73; 83.7)	67.75 (64.95; 70.35)	57.11 (54.48; 59.92)	<0.001
CoA/diaph-Ao	0.8 (0.74; 1.01)	0.88 (0.79; 0.99)	0.98 (0.78; 1.16)	0.153
CoA/mid-Arch	0.85 (0.77; 0.94)	0.86 (0.77; 1.02)	0.97 (0.76; 1.24)	0.198
Residual stenosis, %	19.89 (5.83; 26.07)	15.17 (7.69; 21.11)	14.54 (5.59; 22.22)	0.776
Growth index	0.68 (0.61; 0.75)	0.66 (0.58; 0.75)	0.72(0.65; 0.82)	0.168

Ao—aorta; BSA—body surface area; CoA—coarctation of the aorta; CT scan—computed tomography scan; CoA/diaph-Ao—coarctation of the aorta ratio with aorta at the diaphragmatic level; CoA/mid-Arch—coarctation of the aorta ratio with aortic arch diameter.

**Table 2:** Diagnostic instrumental tests and medications.

	Group 1 (H/W < 0.65) Median (Q1–Q3)	Group 2 (H/W 0.65–0.85) Median (Q1–Q3)	Group 3 (H/W > 0.85) Median (Q1–Q3)	*p*-Value
Non-invasive blood pressure measurements				
Systolic BP RA, mmHg	143.5 (136; 150)	139 (134; 143)	129.5 (118.5; 137)	0.005
Diastolic BP RA, mmHg	83.5 (77; 90)	80 (74; 85)	77.5 (72; 84)	0.061
Systolic BP LA, mmHg	140 (121; 145)	126.5 (120; 140)	126 (116; 135)	0.137
Diastolic BP LA, mmHg	83 (77; 90)	81.5 (74; 85)	78 (73; 83)	0.252
Systolic BP LL, mmHg	151 (130; 161)	147 (135.5; 160.5)	135.5 (125; 148)	0.281
Diastolic BP LL, mmHg	80 (69; 86)	73.5 (1.5; 81.55)	74.55 (70; 84)	0.359
24 h Blood pressure measurements				
Overall systolic BP, mmHg	124 (114.5; 128.5)	118.55 (115; 134)	121 (117.5; 134)	0.919
Overall diastolic BP, mmHg	74 (68.5; 75.5)	74 (69; 76)	65.5 (63.5; 77)	0.261
Systolic dip, %	10.3 (8.95; 18)	9.75 (3.7; 15)	13.2 (6.7; 17.5)	0.448
Diastolic dip, %	18.95 (11.45; 20.6)	10.35 (7.3; 19.2)	16 (13; 19.3)	0.259
Echocardiogram				
LV index, g/m^2^	90.9 (82.11; 106)	86 (75.5; 103)	81.2 (72.3; 97.4)	0.187
LV EF, %	55 (55; 60)	55 (55; 60)	55 (55; 60)	0.552
Gradient CoA, mmHg	19 (13; 30)	20 (12; 28)	22 (14; 32)	0.831
Stiffness				
PWV, m/s	6.1 (5.9; 6.95)	6.2 (5.5; 7)	6.11 (5.9; 77)	0.833
Right CAVI	5.65 (4.95; 6.2)	5.55 (5.22; 6.3)	5.77 (5.22; 6.33)	0.855
Left CAVI	5.7 (5.1; 6.2)	5.9 (5; 6.2)	5.66 (5.2; 6.66)	0.943
Vertebral artery hypoplasia, n (%)	4 (15.3)	10 (37.03)	9 (50)	0.150
RCCA IMT, µm	714 (554; 843)	759 (601; 867)	674 (399; 786)	0.350
LCCA IMT, µm	727 (536; 863)	748 (563.5; 936.5)	653 (555; 807)	0.648
RCCA stiffness	2.94 (2.23; 3.55)	2.56 (2.34; 3.32)	2.92 (2.19; 3.49)	0.905
LCCA stiffness	2.54 (2.15; 3.61)	2.55 (2.05; 3.06)	2.55 (2.07; 3.04)	0.641
Medications				
BB	10 (38.55)	14 (51.99)	5 (27.88)	0.270
ACEI	8 (30.88)	8 (29.66)	4 (22.22)	0.844
ARB	2 (77.7)	1 (3.77)	-	0.619
CCB	4 (15.44)	4 (14.88)	1 (5.66)	0.733
Diuretics	2 (77.7)	-	1 (5.66)	0.355
AB	2 (77.7)	-	-	0.192
Aspirin	-	1 (3.77)	-	1.000
NOAK	2 (7.77)	-	-	0.192
Statins	3 (11.5)	2 (7.4)	-	0.357
Total antihypertensive medications				
0	12 (46.22)	10 (37.37)	11 (61.11)	0.868
1	6 (23.11)	99 (33.3)	4 (22.22)	
2	5 (19.22)	6 (22.22)	2 (11.11)	
3	1 (3.8)	2 (7.4)	1 (5.66)	
4	1 (3.8)	-	-	
5	1 (3.88)	-	-	

AB—alpha-blockers; ACEI—angiotensin-converting enzyme inhibitor; ARB—angiotensin II receptor blocker; BB—beta-blockers; BP—blood pressure; CAVI—cardio–ankle vascular index; CCB—calcium channel blocker; IMT—Intima-Media Thickness; LA—left arm; LCCA—left common carotid artery; LL—lower limb; LV—left ventricle; LV EF—left ventricle ejection fraction; NOAK—novel oral anticoagulant; PWV—pulse wave velocity; RA—right arm; RCCA—right common carotid artery.

**Table 3:** Renal perfusion assessments.

	Group 1 (H/W < 0.65) Median (Q1–Q3)	Group 2 (H/W 0.65–0.85) Median (Q1–Q3)	Group 3 (H/W > 0.85) Median (Q1–Q3)	*p*-Value
Uptake change, %				
Left kidney	2.6 (0.04; 7.69)	0.45 (−3.03; 4.53)	0.13 (−5.47; 3.87)	0.248
Right kidney	−2.42 (−9.49; −0.04)	−0.66 (−4.08; 3.53)	−1.38 (−4.7; 6.34)	0.399
T½ change				
Left kidney	0.19 (−0.06; 0.37)	−0.08 (−0.18; 0.22)	−0.08 (−0.24; 0.09)	0.091
Right kidney	0.2 (−0.06; 0.35)	0.06 (−0.16; 0.31)	−0.06 (−0.23; 0.13)	0.336
T_max_ change				
Left kidney	0.27 (−0.05; 1.13)	0.13 (−0.33; 0.89)	−0.38 (−1.23; 0.43)	0.079
Right kidney	0.61 (−0.02; 2.03)	0.22 (−0.21; 1.65)	0.11 (−1.19; 0.41)	0.229
T_max_/T½, ratio change				
Left kidney	−0.25 (−2.25; 1.75)	0.25 (−1.75; 2)	−0.38 (−1.5; 1.13)	0.638
Right kidney	−1 (−3; 1.5)	−0.25 (−2.13; 1)	−1.25 (−3.13; 1.13)	0.827
30 min/T_max_ ratio change				
Left kidney	0 (−0.03; 0.06)	0.01 (−0.02; 0.05)	0.01 (−0.02; 0.04)	0.947
Right kidney	0 (−0.05; 0.07)	0.01 (−0.03; 0.04)	0.01 (−0.03; 0.03)	0.716
20 min/3 min ratio change				
Left kidney	0 (−0.03; 0.06)	−0.01 (−0.02; 0.06)	−0.02 (−0.04; 0.02)	0.267
Right kidney	0 (−0.06; 0.08)	0 (−0.04; 0.03)	−0.03 (−0.11; −0.01)	0.052

T_max_ = time to the maximum counts; T½ = time to the half-peak counts; 30 min/max = ratio of the renal counts at 30 min to the maximum counts; 30 min/3 min = ratio of the counts at 30 to the 3 min counts.

## Data Availability

Data are available in Vilnius University Hospital Santaros Clinics’ database.
